# The genome sequence of the Golden-tailed Leafwalker, *Xylota sylvarum* (Linnaeus, 1758)

**DOI:** 10.12688/wellcomeopenres.19241.1

**Published:** 2023-03-23

**Authors:** Liam M. Crowley, Will Nash, University of Oxford and Wytham Woods Genome Acquisition Lab, Darwin Tree of Life Barcoding collective, Wellcome Sanger Institute Tree of Life programme, Wellcome Sanger Institute Scientific Operations: DNA Pipelines collective, Tree of Life Core Informatics collective, Darwin Tree of Life Consortium

**Affiliations:** 1 Department of Biology, University of Oxford, Oxford, England, UK; 2 Earlham Institute, Norwich, England, UK

**Keywords:** Xylota sylvarum, Golden-tailed Leafwalker, genome sequence, chromosomal, Diptera

## Abstract

We present a genome assembly from an individual male *Xylota sylvarum* (the Golden-tailed Leafwalker; Arthropoda; Insecta; Diptera; Syrphidae). The genome sequence is 534.8 megabases in span. Most of the assembly is scaffolded into five chromosomal pseudomolecules, including the assembled X sex chromosome. The mitochondrial genome has also been assembled and is 16.0 kilobases in length. Gene annotation of this assembly on Ensembl identified 11,993 protein coding genes.

## Species taxonomy

Eukaryota; Metazoa; Ecdysozoa; Arthropoda; Hexapoda; Insecta; Pterygota; Neoptera; Endopterygota; Diptera; Brachycera; Muscomorpha; Syrphoidea; Syrphidae; Eristalinae; Xylotini; *Xylota*; *Xylota sylvarum* (Linnaeus, 1758) (NCBI:txid374264).

## Background

*Xylota sylvarum* (Linnaeus, 1758) is a large, black hoverfly with an elongate body and a characteristic covering of golden yellow hairs on tergite 4, at the tip of its abdomen ( Stubbs & Falk, 2002). In the field, it can be readily confused with *X. xanthocnema* (Collin, 1939) ( Ball & Morris, 2015; Stubbs & Falk, 2002). These two species are separated on the basis of the colour of the hind tibiae: Those of *X. sylvarum* are dark at the distal end ( Ball & Morris, 2015; Stubbs & Falk, 2002; van Veen, 2004), whereas the tibia of *X. xanthocnema* are yellow. Care is required as *X. sylvarum* can be misidentified if viewed from above. According to ( Rotheray, 1993; Rotheray, 2004), the larvae can also be separated if examined in detail.

*Xylota sylvarum* is abundant across the UK and Europe ( GBIF Secretariat, 2022), being listed as ‘Least Concern’ ( Vujić *et al.*, 2022). It is widespread in England and Wales, but scarcer in Scotland ( Ball *et al.*, 2011). Larvae have been reared from a wide range of rotting tree material from a variety of species, both broadleaved and coniferous ( Hartley, 1961; Hartley, 1963; Rotheray, 2004). Adults, which fly May-October, are mostly seen upon foliage ( Ball & Morris, 2015; Stubbs & Falk, 2002). Adults feed on windblown pollen stuck to aphid honeydew on leaf surfaces ( Ssymank & Gilbert, 1993), although will also visit flowers ( Ball & Morris, 2015). The chromosome-level genome assembly presented here is, to our knowledge, the first high-quality resource developed for a member of the genus *Xylota*.

### Genome sequence report

**Figure 1.**
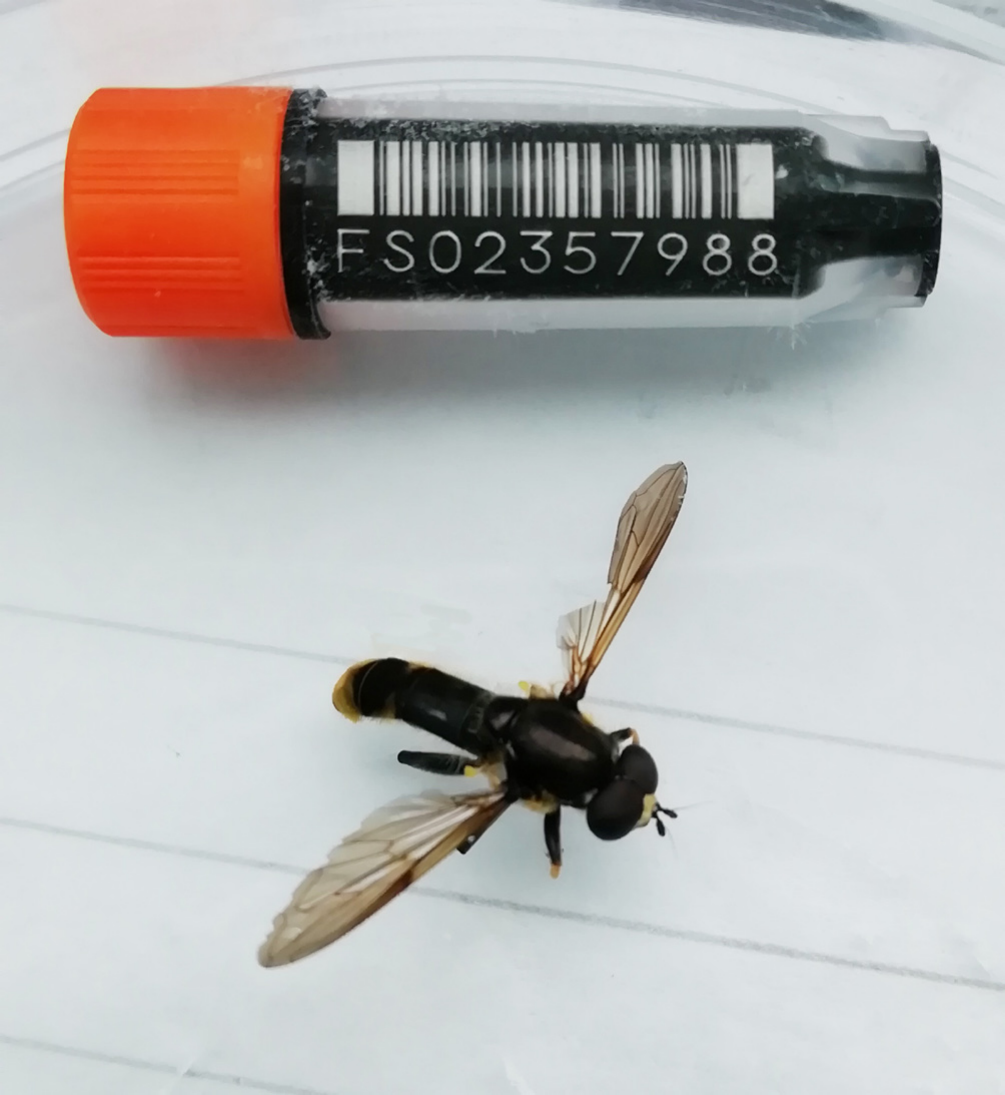
Photograph of the *Xylota sylvarum* (idXylSylv2) specimen used for genome sequencing.

The genome was sequenced from one male *Xylota sylvarum* ( Figure 1) collected from Wytham Woods, Oxfordshire (biological vice-county: Berkshire), UK (latitude 51.78, longitude –1.33). A total of 34-fold coverage in Pacific Biosciences single-molecule HiFi long reads and 48-fold coverage in 10X Genomics read clouds were generated. Primary assembly contigs were scaffolded with chromosome conformation Hi-C data. Manual assembly curation corrected 37 missing joins or mis-joins and removed six haplotypic duplications, reducing the assembly length 0.86% and the scaffold number by 24.2%, and increasing the scaffold N50 by 327.13%.

**Figure 2.**
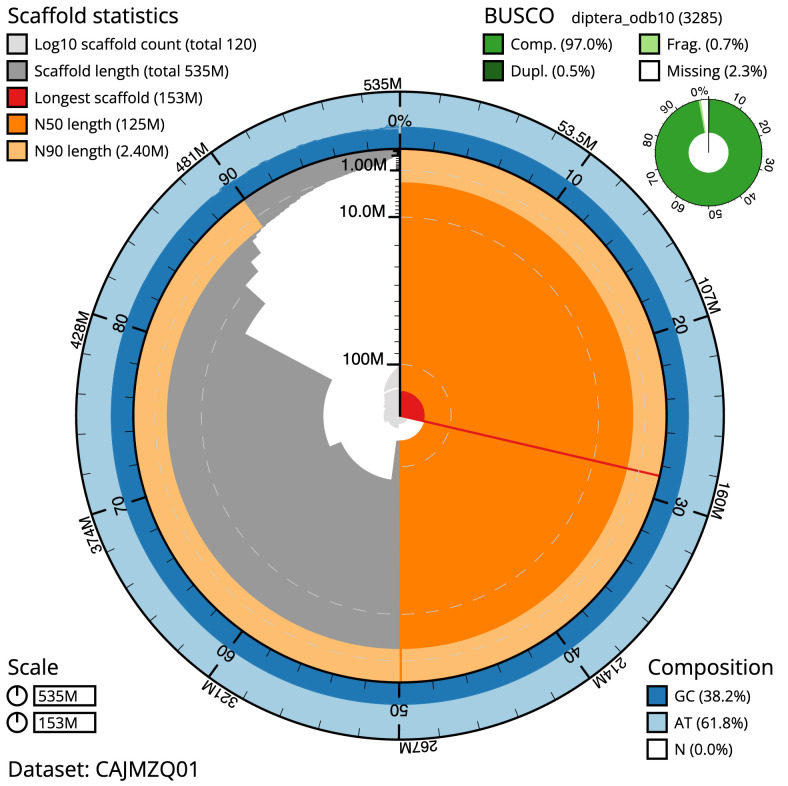
Genome assembly of *Xylota sylvarum*, idXylSylv2.1: metrics. The BlobToolKit Snailplot shows N50 metrics and BUSCO gene completeness. The main plot is divided into 1,000 size-ordered bins around the circumference with each bin representing 0.1% of the 534,835,910 bp assembly. The distribution of scaffold lengths is shown in dark grey with the plot radius scaled to the longest scaffold present in the assembly (153,043,062 bp, shown in red). Orange and pale-orange arcs show the N50 and N90 scaffold lengths (124,801,819 and 2,404,465 bp), respectively. The pale grey spiral shows the cumulative scaffold count on a log scale with white scale lines showing successive orders of magnitude. The blue and pale-blue area around the outside of the plot shows the distribution of GC, AT and N percentages in the same bins as the inner plot. A summary of complete, fragmented, duplicated and missing BUSCO genes in the diptera_odb10 set is shown in the top right. An interactive version of this figure is available at https://blobtoolkit.genomehubs.org/view/Xylota%20sylvarum/dataset/CAJMZQ01/snail.

The final assembly has a total length of 534.8 Mb in 119 sequence scaffolds with a scaffold N50 of 124.8 Mb ( Table 1). Most (87.26%) of the assembly sequence was assigned to five chromosomal-level scaffolds, representing four autosomes, and the X sex chromosome. Chromosome-scale scaffolds confirmed by the Hi-C data are named in order of size ( Figure 2– Figure 5; Table 2). While not fully phased, the assembly deposited is of one haplotype. Contigs corresponding to the second haplotype have also been deposited. The estimated *k*-mer-based Quality Value (QV) of the final assembly is 56.1 with *k*-mer based completeness of 99.99%, and the assembly has a BUSCO v5.3.2 ( Manni *et al.*, 2021) completeness of 97.0% (single = 96.5%, duplicated = 0.5%), using the diptera_odb10 reference set ( *n* = 3,285).

**Table 1.** Genome data for *Xylota sylvarum*, idXylSylv2.1.

Project accession data		
Assembly identifier	idXylSylv2.1	
Species	*Xylota sylvarum*	
Specimen	idXylSylv2	
NCBI taxonomy ID	374264	
BioProject	PRJEB42947	
BioSample ID	SAMEA7520173	
Isolate information	idXylSylv2, male	
Assembly metrics *		*Benchmark*
Consensus quality (QV)	56.1	*≥ 50*
*k*-mer completeness	99.99%	*≥ 95%*
BUSCO **	C:97.0%[S:96.5%,D:0.5%], F:0.7%,M:2.3%,n:3,285	*C ≥ 95%*
Percentage of assembly mapped to chromosomes	87.26%	*≥ 95%*
Sex chromosomes	X chromosome	*localised homologous pairs*
Organelles	Mitochondrial genome assembled	*complete single alleles*
Raw data accessions		
PacificBiosciences SEQUEL II	ERR6635595	
10X Genomics Illumina	ERR6054383–ERR6054386	
Hi-C Illumina	ERR6054387	
PolyA RNA-Seq Illumina	ERR6054388	
Genome assembly		
Assembly accession	GCA_905220385.1	
*Accession of alternate haplotype*	GCA_905220405.1	
Span (Mb)	534.8	
Number of contigs	182	
Contig N50 length (Mb)	29.2	
Number of scaffolds	119	
Scaffold N50 length (Mb)	124.8	
Longest scaffold (Mb)	153.0	
Genome annotation		
Number of protein-coding genes	11,993	
Number of non-coding genes	1,160	
Number of gene transcripts	19,577	

* Assembly metric benchmarks are adapted from column VGP-2020 of “Table 1: Proposed standards and metrics for defining genome assembly quality” from ( Rhie *et al.*, 2021). ** BUSCO scores based on the diptera_odb10 BUSCO set using v5.3.2. C = complete [S = single copy, D = duplicated], F = fragmented, M = missing, n = number of orthologues in comparison. A full set of BUSCO scores is available at https://blobtoolkit.genomehubs.org/view/Xylota%20sylvarum/dataset/CAJMZQ01/busco.

**Figure 3.**
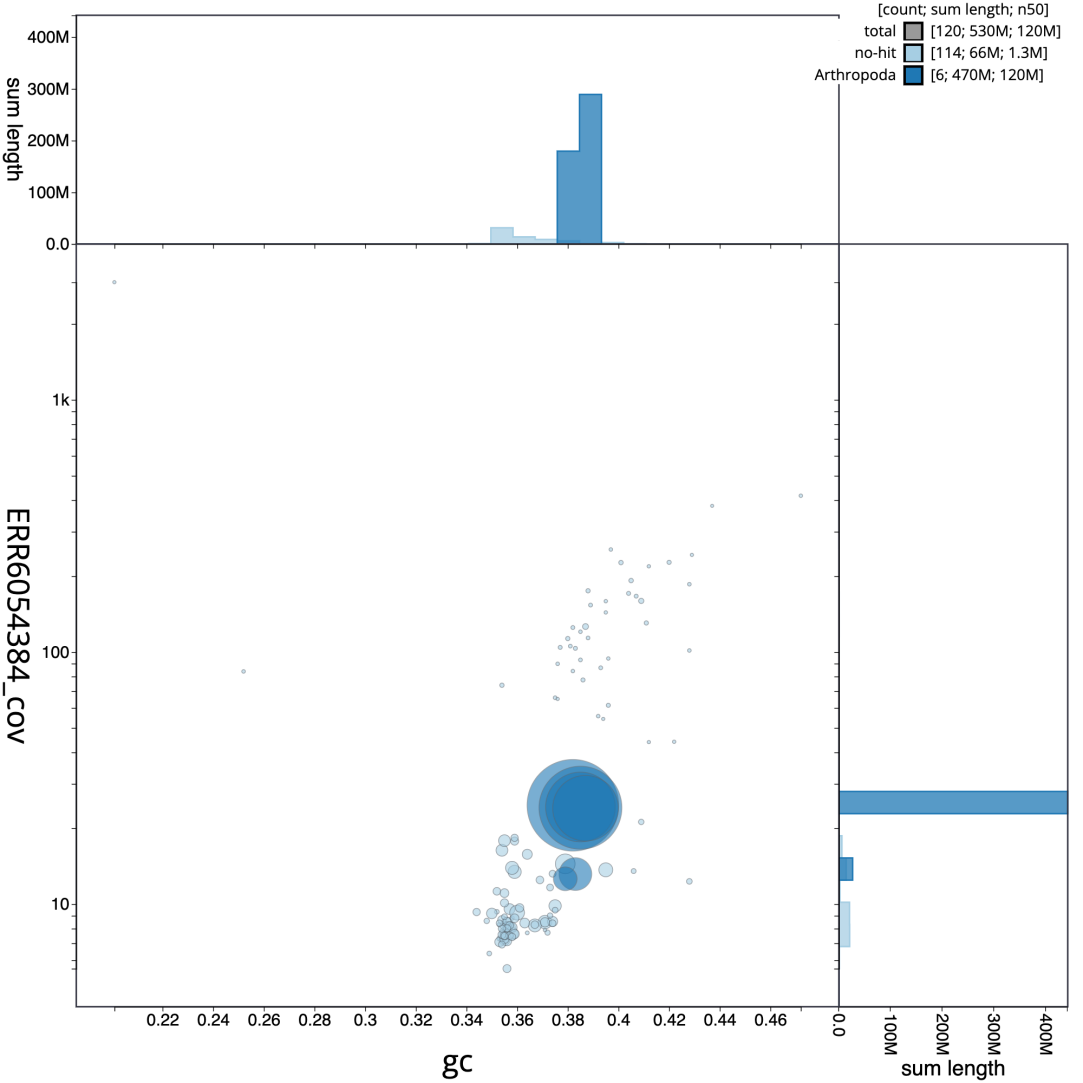
Genome assembly of *Xylota sylvarum*, idXylSylv2.1: GC coverage. BlobToolKit GC-coverage plot. Scaffolds are coloured by phylum. Circles are sized in proportion to scaffold length. Histograms show the distribution of scaffold length sum along each axis. An interactive version of this figure is available at https://blobtoolkit.genomehubs.org/view/Xylota%20sylvarum/dataset/CAJMZQ01/blob.

**Figure 4.**
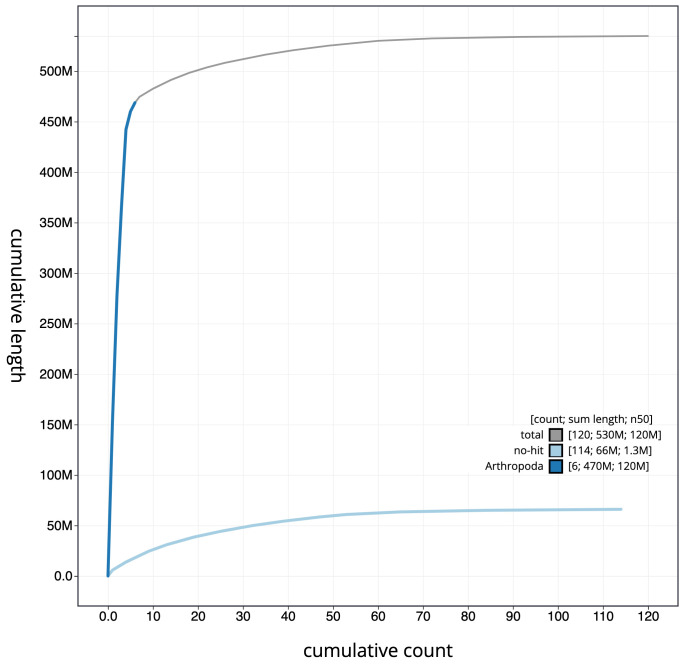
Genome assembly of *Xylota sylvarum*, idXylSylv2.1: cumulative sequence. BlobToolKit cumulative sequence plot. The grey line shows cumulative length for all scaffolds. Coloured lines show cumulative lengths of scaffolds assigned to each phylum using the buscogenes taxrule. An interactive version of this figure is available at https://blobtoolkit.genomehubs.org/view/Xylota%20sylvarum/dataset/CAJMZQ01/cumulative.

**Figure 5.**
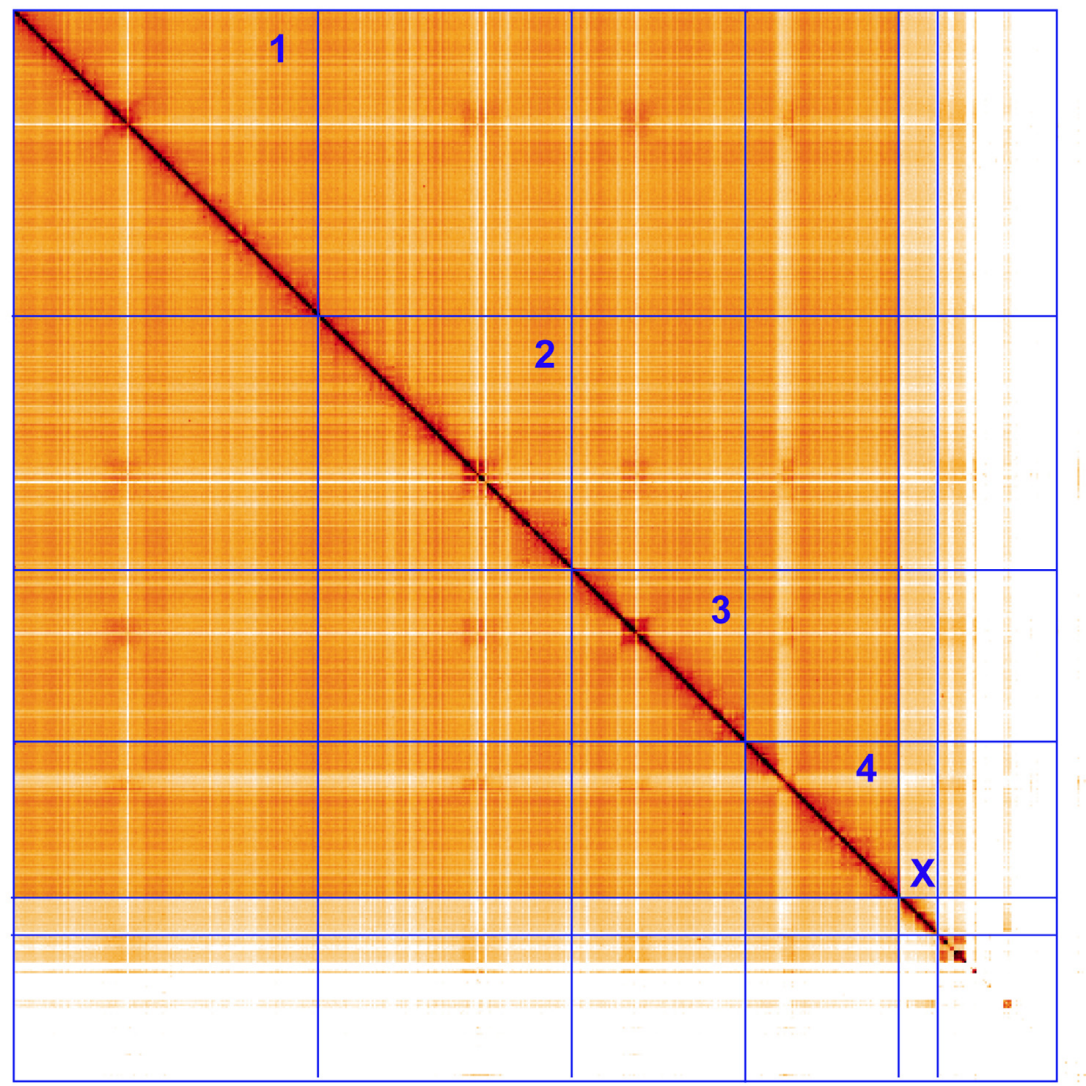
Genome assembly of *Xylota sylvarum*, idXylSylv2.1: Hi-C contact map. Hi-C contact map of the idXylSylv2.1 assembly, visualised using HiGlass. Chromosomes are shown in order of size from left to right and top to bottom. An interactive version of this figure may be viewed at https://genome-note-higlass.tol.sanger.ac.uk/l/?d=RfSk_MelQaWkABlgTka5NA.

**Table 2.** Chromosomal pseudomolecules in the genome assembly of *Xylota sylvarum*, idXylSylv2.

INSDC accession	Chromosome	Size (Mb)	GC%
LR999957.1	1	153.04	38.2
LR999958.1	2	124.8	38.5
LR999959.1	3	87.31	38.5
LR999960.1	4	77.11	38.7
LR999961.1	X	17.73	38.3
LR999962.1	MT	0.02	19.4
-	unplaced	74.82	36.6

### Genome annotation report

The *X. sylvarum* genome assembly (GCA_905220385.1) was annotated using the Ensembl rapid annotation pipeline ( Table 1; https://rapid.ensembl.org/Xylota_sylvarum_GCA_905220385.1/). The resulting annotation includes 19,577 transcribed mRNAs from 11,993 protein-coding and 1,160 non-coding genes.

## Methods

### Sample acquisition and nucleic acid extraction

Two male *Xylota sylvarum* specimens (idXylSylv1 and idXylSylv2) were collected from Wytham Woods, Oxfordshire (biological vice-county: Berkshire), UK (latitude 51.78, longitude –1.33) on 8 August 2019 and 20 August 2019. The specimens were taken from woodland habitat by Liam Crowley (University of Oxford) by netting. The specimens were identified by the collector and snap frozen on dry ice.

DNA was extracted at the Tree of Life laboratory, Wellcome Sanger Institute (WSI). The idXylSylv2 sample was weighed and dissected on dry ice with tissue set aside for Hi-C sequencing. Thorax tissue was disrupted using a Nippi Powermasher fitted with a BioMasher pestle. High molecular weight (HMW) DNA was extracted using the Qiagen MagAttract HMW DNA extraction kit. Low molecular weight DNA was removed from a 20 ng aliquot of extracted DNA using the 0.8X AMpure XP purification kit prior to 10X Chromium sequencing; a minimum of 50 ng DNA was submitted for 10X sequencing. HMW DNA was sheared into an average fragment size of 12–20 kb in a Megaruptor 3 system with speed setting 30. Sheared DNA was purified by solid-phase reversible immobilisation using AMPure PB beads with a 1.8X ratio of beads to sample to remove the shorter fragments and concentrate the DNA sample. The concentration of the sheared and purified DNA was assessed using a Nanodrop spectrophotometer and Qubit Fluorometer and Qubit dsDNA High Sensitivity Assay kit. Fragment size distribution was evaluated by running the sample on the FemtoPulse system.

RNA was extracted from head and thorax tissue of idXylSylv1 in the Tree of Life Laboratory at the WSI using TRIzol, according to the manufacturer’s instructions. RNA was then eluted in 50 μl RNAse-free water and its concentration assessed using a Nanodrop spectrophotometer and Qubit Fluorometer using the Qubit RNA Broad-Range (BR) Assay kit. Analysis of the integrity of the RNA was done using Agilent RNA 6000 Pico Kit and Eukaryotic Total RNA assay.

### Sequencing

Pacific Biosciences HiFi circular consensus and 10X Genomics read cloud DNA sequencing libraries were constructed according to the manufacturers’ instructions. Poly(A) RNA-Seq libraries were constructed using the NEB Ultra II RNA Library Prep kit. DNA and RNA sequencing was performed by the Scientific Operations core at the WSI on Pacific Biosciences SEQUEL II (HiFi), Illumina HiSeq 4000 (RNA-Seq) and HiSeq X Ten (10X) instruments. Hi-C data were also generated from abdomen tissue of idXylSylv2 using the Arima v1 kit and sequenced on the HiSeq X Ten instrument.

### Genome assembly

Assembly was carried out with Hifiasm ( Cheng *et al.*, 2021) and haplotypic duplication was identified and removed with purge_dups ( Guan *et al.*, 2020). One round of polishing was performed by aligning 10X Genomics read data to the assembly with Long Ranger ALIGN, calling variants with FreeBayes ( Garrison & Marth, 2012). The assembly was then scaffolded with Hi-C data ( Rao *et al.*, 2014) using SALSA2 ( Ghurye *et al.*, 2019). The assembly was checked for contamination and corrected using the gEVAL system ( Chow *et al.*, 2016) as described previously ( Howe *et al.*, 2021). Manual curation was performed using gEVAL, HiGlass ( Kerpedjiev *et al.*, 2018) and Pretext ( Harry, 2022). The mitochondrial genome was assembled using MitoHiFi ( Uliano-Silva *et al.*, 2022), which performed annotation using MitoFinder ( Allio *et al.*, 2020).

To evaluate the assembly, MerquryFK was used to estimate *k-*mer completeness and consensus quality (QV) ( Rhie *et al.*, 2020). The genome was analysed and BUSCO scores ( Simão *et al.*, 2015) were generated within the BlobToolKit environment ( Challis *et al.*, 2020. Table 3 contains a list of software tool versions and sources.

**Table 3.** Software tools and versions used.

Software tool	Version	Source
BlobToolKit	3.5.2	https://github.com/blobtoolkit/blobtoolkit
BUSCO	5.3.2	https://gitlab.com/ezlab/busco
FreeBayes	1.3.1-17- gaa2ace8	https://github.com/freebayes/freebayes
gEVAL	N/A	https://geval.org.uk/
Hifiasm	0.12	https://github.com/chhylp123/hifiasm
HiGlass	1.11.6	https://github.com/higlass/higlass
Long Ranger ALIGN	2.2.2	https://support.10xgenomics.com/genome-exome/software/pipelines/latest/advanced/other- pipelines
Merqury	MerquryFK	https://github.com/thegenemyers/MERQURY.FK
MitoHiFi	2	https://github.com/marcelauliano/MitoHiFi
PretextView	0.2	https://github.com/wtsi-hpag/PretextView
purge_dups	1.2.3	https://github.com/dfguan/purge_dups
SALSA	2.2	https://github.com/salsa-rs/salsa

### Genome annotation

The Ensembl gene annotation system ( Aken *et al.*, 2016) was used to generate annotation for the *Xylota sylvarum* assembly (GCA_905220385.1). Annotation was created primarily through alignment of transcriptomic data to the genome, with gap filling via protein to-genome alignments of a select set of proteins from UniProt ( UniProt Consortium, 2019).

### Ethics and compliance issues

The materials that have contributed to this genome note have been supplied by a Darwin Tree of Life Partner. The submission of materials by a Darwin Tree of Life Partner is subject to the Darwin Tree of Life Project Sampling Code of Practice. By agreeing with and signing up to the Sampling Code of Practice, the Darwin Tree of Life Partner agrees they will meet the legal and ethical requirements and standards set out within this document in respect of all samples acquired for, and supplied to, the Darwin Tree of Life Project. All efforts are undertaken to minimise the suffering of animals used for sequencing. Each transfer of samples is further undertaken according to a Research Collaboration Agreement or Material Transfer Agreement entered into by the Darwin Tree of Life Partner, Genome Research Limited (operating as the Wellcome Sanger Institute), and in some circumstances other Darwin Tree of Life collaborators.

## Data Availability

European Nucleotide Archive: Xylo *ta sylvarum* (golden-tailed hoverfly). Accession number PRJEB42947; https://identifiers.org/ena.embl/PRJEB42947. ( Wellcome Sanger Institute, 2021) The genome sequence is released openly for reuse. The *Xylota sylvarum* genome sequencing initiative is part of the Darwin Tree of Life (DToL) project. All raw sequence data and the assembly have been deposited in INSDC databases. Raw data and assembly accession identifiers are reported in Table 1.

## Author information

Members of the University of Oxford and Wytham Woods Genome Acquisition Lab are listed here: https://doi.org/10.5281/zenodo.4789928.

Members of the Darwin Tree of Life Barcoding collective are listed here: https://doi.org/10.5281/zenodo.4893703.

Members of the Wellcome Sanger Institute Tree of Life programme are listed here: https://doi.org/10.5281/zenodo.4783585.

Members of Wellcome Sanger Institute Scientific Operations: DNA Pipelines collective are listed here: https://doi.org/10.5281/zenodo.4790455.

Members of the Tree of Life Core Informatics collective are listed here: https://doi.org/10.5281/zenodo.5013541.

Members of the Darwin Tree of Life Consortium are listed here: https://doi.org/10.5281/zenodo.4783558.
