# Supplementary material for: The genome sequence of the Golden-tailed Leafwalker, Xylota sylvarum (Linnaeus, 1758)
Source: Wellcome Open Res. 2023 Mar 23;8:134. [Version 1] doi: 10.12688/wellcomeopenres.19241.1 (PMC11066536; doi:10.12688/wellcomeopenres.19241.1)
Supplement: Peer review history

# Reviewer report: Reviewer response for version 1

**DOI:** 10.21956/wellcomeopenres.21324.r78631

**Published:** 2024-05-02

**Authors:** Frederique Hilliou

**Affiliations:** 1 Institut Sophia Agrobiotech, Institut national de la recherche pour l'agriculture, l'alimentation et l'environnement, Sophia Antipolis, France; 2 Universite Cote d'Azur, Nice, Provence-Alpes-Côte d'Azur, France

The manuscript reports the genome assembly from an individual male *Xylota sylvarum* (the Golden-tailed Leafwalker; Arthropoda; Insecta; Diptera; Syrphidae) at a level with 5 chromosomal pseudomolecules including the assembled X sex chromosome. The mitochondrial genome of *Xylota sylvarum*  was also assembled. The manuscript provides useful morphological descriptions of *Xylota sylvarum*  as well as the geographical distribution in Europe. The genome sequence is released openly for reuse.

 As a minor revision I suggest to update the manuscript as other *Xylota* genomes are available: *Xylota segnis* (ID138115), GCA _963583995.1 and GCA_963583945.1. I also suggest to the authors to explain what is a full phased assembly of a genome.

 Once the revision done, since the authors clearly provide a clear description of the insect as well as the methodology followed to obtained the genome, I approve the publication. This work is an important addition to the genome database and will be a useful addition to many study on insect phylogeny for instance.

Are sufficient details of methods and materials provided to allow replication by others?

Yes

Is the rationale for creating the dataset(s) clearly described?

Yes

Are the datasets clearly presented in a useable and accessible format?

Yes

Are the protocols appropriate and is the work technically sound?

Yes

Reviewer Expertise:

insect genomics, detoxification, insect evolution and adaptation to their environment

I confirm that I have read this submission and believe that I have an appropriate level of expertise to confirm that it is of an acceptable scientific standard.

# Reviewer report: Reviewer response for version 1

**DOI:** 10.21956/wellcomeopenres.21324.r78634

**Published:** 2024-04-26

**Authors:** Maria Cristina Arias

**Affiliations:** 1 University of Sao Paulo, Sao Paulo, Brazil

The authors report here the genome assembly for Xylota sylvarum (Diptera). The total genome size reached 534.8 Mb and is organized in 119 scaffolds  (N50 of 124.8 Mb). The Hi-C data enabled to ensure the assignment of those scaffolds at chromosomal level (4 autosomes and the X sex chromosome), Through Ensembl rapid annotation pipeline it was possible to identify 19,577 transcripts from 11,993 protein coding regions. The note is written according to the template format of the Journal and the data will be valuable for further studies. As a minor revision I suggest clarifying the differences found between the number of predicted genes (12,000) and the number estimated by using the transcriptome data. Are you referring to possible isoforms?  Also the mitochondrial genome is reported as 16 kb (Abstract) but in table 2 (MT)  the size reported is 20 kb. So please clarify the mtGenome size.

Are sufficient details of methods and materials provided to allow replication by others?

No

Is the rationale for creating the dataset(s) clearly described?

Yes

Are the datasets clearly presented in a useable and accessible format?

Yes

Are the protocols appropriate and is the work technically sound?

Yes

Reviewer Expertise:

Genetics and Evolution of bees

I confirm that I have read this submission and believe that I have an appropriate level of expertise to confirm that it is of an acceptable scientific standard.
